# Macroeconomic effects of crude oil shocks: Evidence from South Asian countries

**DOI:** 10.3389/fpsyg.2022.967643

**Published:** 2022-08-15

**Authors:** Iftikhar Ahmad, Shahid Iqbal, Salim Khan, Heesup Han, Alejandro Vega-Muñoz, Antonio Ariza-Montes

**Affiliations:** ^1^Riphah School of Leadership, Faculty of Management Science (FMS), Riphah International University, Islamabad, Pakistan; ^2^College of Hospitality and Tourism Management, Sejong University, Seoul, South Korea; ^3^Public Policy Observatory, Universidad Autónoma de Chile, Santiago, Chile; ^4^Social Matters Research Group, Universidad Loyola Andalucía, Córdoba, Spain

**Keywords:** crude oil, macroeconomic indicators, interest rate, exchange rate, South Asian countries, green energy sources

## Abstract

This research tends to convey the relationship between crude oil price volatility and key macroeconomics indicators, i.e., gross domestic product (GDP), inflation rate (IR), interest rate, and exchange rate. The study collected the time-series data (2000–2020) from the South Asian countries (Afghanistan, Bangladesh, Bhutan, India, Maldives, Nepal, Pakistan, and Sri Lanka), and employed macroeconomic policy modeling tools (impulse response function and the prediction error variance decomposition technique) in the vector autorepression (VAR) setup. The outcome of the impulse response function explained considerable variance among macroeconomic indicators in response to crude oil price shocks. The macroeconomic indicators are extremely susceptible to minor fluctuations in oil prices causing a significant impact on the region's socioeconomic situation. The result of variance decomposition indicates that each country in the region reacts differently to crude oil price volatility which reflects their macroeconomics fundamentals, independent policy, sector structure, and country differences. The findings support change in public policies in a way to reduce their dependency on oil energy and encourage them toward renewal and green energy sources for better environmental results and sustainable development.

## Introduction

Since the mid-1950's, crude oil has been regarded as the world's most significant source of energy, serving as the lifeblood and engine of the industrialized world and becoming a vital source of economic growth for many countries (Mehmood et al., 2021). It is also considered a crucial strategic source in world politics (Shao and Hua, 2022). Its products are vital to human civilizations because they are required for a number of activities in daily life ranging from industry to routine household activities. Volatility in the price of oil could have a significant macroeconomic impact on both exporting and importing countries. It also affects transportation, production costs, and heating bills which generate uncertainty in the world economy (Abdelsalam, 2020). Oil price volatility is a key component and important commodity of the global economic system and is regarded as one of the most important factors of the national and international economic progress. Therefore, the crude oil market's supply and demand have a substantial influence on the global currency (Nyangarika et al., 2018). Because of such immense significance, the price of oil products is more volatile and unpredictable than that of any other commodity. The prices of crude oil directly affect industrial output, investment, international commerce, and other production forms and even decrease in family purchasing power due to inelasticity of demand. Furthermore, the high price of crude oil directly influences consumer products and services, creating future uncertainty. It also has a detrimental impact on economic performance as it diverts spending away from capital goods and big-ticket purchases. Volatility in oil prices has a varying effect on importing and exporting countries. The economy of exporting countries heavily depends on oil revenue. An increase in oil prices led to more resources for developmental projects. Therefore, changes in oil prices have a significant impact on how well monetary and fiscal policies function (Saddiqui et al., 2018). Crude oil is a necessary input for industrial goods. Its volatility also affects the exchange rate and substantially influences unemployment and investment (Rafiq et al., 2009). Moreover, the rise in the crude oil prices affects the interest rate and consumer expenditure resulting in inflation. Increased consumer spending reduces savings, which in turn, affects capital creation. Consequently, manufacturing cost increases and firm production decreases resulting in a huge drop in overall output and retarding economic progression (Choi et al., 2018). Hence, fluctuations in oil prices influence all business activities, both, local and as well as international (Nazariyan and Amiri, 2014; Adam, 2016). The valuable and positive influence of the oil price instability is more substantial than the negative impact. Zakaria et al. (2021) reported that the oil prices and inflation rate are co-integrated and that the price of oil Granger affects inflation. Similarly, the impulse response function indicated that a rise in oil prices has a considerable and long-term influence on the inflation rate. Therefore, according to the results of variance decomposition, a rise in oil prices explains a change in the inflation rate in the long term. The instability in crude oil prices is affected by various factors such as a change in industrial production, economic growth, exchange rate variation, political aspects, financial speculation, financial crises, and reduction in oil prices due to low demand or high supply. Accordingly, this study aims to examine the impact of oil prices on macro-economic factors such as GDP, inflation, interest, and exchange rate. By focusing on the South-Asian region, the study will help the policymakers to realize the role of oil prices in the macro-economic indicators of these countries that are largely dependent on the import of crude oil. This study contributes to the body of literature in two ways. First, it examines the impact of oil price volatility on the economy of the South-Asian countries. To our knowledge, no prior study considered this point and selected this region before. Second, it examines what extent the effect of oil prices is asymmetrical. In this study, attempts have been made to investigate whether and how much oil prices and its volatility should be considered when calculating and forecasting economic growth. The rest of the article is as structured as: The literature review is given in part 2, data and the model are described in Section 3, the empirical findings are presented in Section 4, and the conclusion of the study is in Section 5.

## Literature review

As per the widely accepted theory of economic growth developed by Samuelson and Nordhouse (1985) production is reliant on energy. The theory assumes that production is made possible by fundamental factors such as land, labor, and capital and also by the intermediate factors such as coal, fuel, oil, and gas. Second, the symmetric/linear relationship growth theory developed by Hamilton (1983) and Hooker (1986) assumes the significant inverse relationship between GDP growth and oil price hikes. Other studies (Bruno and Sachs, 1982; Darby, 1982) also found a negative relationship between hike in oil prices and economic growth. The sudden hike in prices in 1970 and subsequent economic recessions due to low oil supply gained considerable attention. Oil is the main source of energy for economic progression and its crises may jeopardize world political and economic sustainability. According to Mo et al. (2019) increase in oil prices stimulates economic growth in the long run while the negative effect is only observed in the short run. According to the study by Cheng et al. (2019), an escalation in oil prices reduces real GDP and investment while the reduction in oil prices boosts the macroeconomy. Uncertainty in oil prices has a significantly negative impact on real GDP and investment (Nguyen et al., 2020). According to Umar et al. (2020), energy is the driving force behind economic growth. Oil, which accounts for one-third of global energy consumption, is regarded as the primary energy source. Miamo and Achuo (2021) found a two-way causal relationship between GDP and crude oil prices. They recommended that to stimulate economic growth, the government should spend more on human capital, promote economic diversification, expand the oil sector, manage oil revenues effectively, and step-up efforts to combat corruption. Iyke (2019) studied the uncertainty of crude oil prices and production output in Nigeria and reported that an increase in oil prices leads to decrease in the actual level of production. Zulfigarov and Neuenkirch (2020) studied the relationship between fluctuations in oil prices and economic activities in Azerbaijan by utilizing the vector autoregressive statistical model for the period 2002–2018. They revealed that innovation in oil prices leads to higher inflation, and other macroeconomic variables responded differently to price volatility. Yildirim and Arifli (2021) studied the effects of oil price shocks on the economy of Azerbaijan by employing the VAR model during 2006–2018. They found that a negative oil price shock declines trade balance, depreciates the currency, increases inflation, and decreases overall economic activities.

Phan et al. (2020) investigated and reported a conflicting relationship between uncertainty in crude oil prices and the performance of firms at the sectoral and aggregate levels. Azad and Serletis (2020) examined the impact of oil price volatility on economic activity by using the statistical models. They revealed that uncertainty in oil prices has a significant impact on the GDP of the seven-emerging market (EM7) economies. They also found that uncertainty in oil prices negatively affects the world crude oil production. Jiang and Liu (2021) utilized the NRDL model and revealed that uncertainty in crude oil prices has an asymmetric impact on shock prices. Khan et al. (2020) studied the relationship between industrial production with crude oil and natural gas prices by using the wavelet-based quantile regression model. They revealed that crude oil has a positive, and natural gas has a negative relationship with industrial production in the short term. Aloui et al. (2018) revealed a positive and non-homogeneous relationship between oil prices and production growth in Saudi Arabia by utilizing a Novel Wavelet approach. Balashova and Serletis (2020) reported a positive and statistically significant relationship between the industrial production index and oil prices in Russian Federation reported a regime-switching relationship between gas and crude oil prices (Atil et al., 2014; Brigida, 2014).

Besso et al. (2017) used a panel VAR model and concluded that oil price has a negative and significant long run effect on GDP growth. Although, the result of the study is consistent with a previous study (Omolade et al., 2019) and consistent with the finding of Omojolaibi and Egwaikhide (2013). A study conducted by Nusair (2016) revealed a positive and significant relationship between gross domestic product and oil prices. Zhang and Tu (2016) found a significant and symmetric impact of oil prices on China's Metal market production. According to Zamani (2016), natural gas prices and crude oil prices move in the same direction. The irregular characteristics were reported in the relationship of both oil and gas price movement (Serletis and Rangel-Ruiz, 2004; Ramberg and Parsons, 2012). Omojolaibi and Egwaikhide (2013) studied the impact of crude oil fluctuations on GDP growth and inflation rate by selecting United State (US), China, and Japan and claims that an increase in oil prices inversely affects the GDP growth in China and is positive on the GDP growth of US and Japan. They concluded that the impact of oil price fluctuation on the GDP growth rate is slower for developed net oil importing economies such as the US and Japan than on an emerging economy such as China. The study by Hamilton (2008) reported a conflicting relationship between economic growth and oil prices. The influence of oil price shocks on economic development depends on the intensity and source of oil shock (Kilian, 2009). These and other similar studies provide inconsistent findings making it too hard for the research community to draw a conclusive inference. Besides, most of these previous studies have been conducted in a limited context (i.e., specifying one or two countries), thereby limiting their generalizability. Therefore, this study aims to investigate the asymmetric behavior of the South Asian countries with respect to change in crude oil prices in different economic cycles. The research question that this study aims to answer is:

1. Is the South Asian economy behaving differently toward the price shock of crude oil over the economic cycle?

While the method used to answer the research, this study employed the statistical methods of the VAR modeling to examine the relationship between crude oil price volatility and macroeconomic indicators of the South Asian countries in different time horizons.

## Methodology and materials

This section of the research article provides a detailed description of the research methodology. It offers data collection, data instruments, and research variables.

### Data source and variables

The study aims to investigate the association between crude oil prices and macroeconomic indicators that is GDP, inflation rate, interest rate, and exchange rate. To analyze this relation, last 21 years (2000–2020) of the selected variables are extracted from the World Bank.

### The econometrics framework

This study utilizes E-views 12 to incorporate vector autoregressive (VAR) model, to analyze the crude oil price shocks on the GDP, inflation rate, interest rate, and exchange rate in South Asian countries (Afghanistan, Bangladesh, Bhutan, India, Maldives, Nepal, Pakistan, and Sri Lanka). The study variables are given in Table 1. Vector autoregression (VAR), as an increasingly popular statistical model, was used to forecast the relationship between multiple quantities as they change over time as well as for economic analysis. Different tools have been proposed by experts such as impulse response analysis, forecast error decompositions, historical decomposition, and the analysis of forecast scenarios for separating the relationship between the variables in the VAR model.

**Table 1:** Study variables.

**Variables**	**Time period**	**Illustration**
Crude oil	2000 to 2020	Annual closing price of the crude oil in international market
GDP	Current $ U.S.	Real Gross domestic product growth in percentage.
Inflation	base year 2010	Annul Consumer Price Index (CPI) data of the individual country is used to measure the inflation.
Interest rate		Annual real rate of interest (%) of each country is used to measure this variable.
Exchange rate		Annual real exchange rate of individual country against US $

The standard VAR is a reduced form of model which is linked to an economic model, where economic interpretation of the result is often impossible. If the economic theory is used to provide a relationship between forecast errors and fundamental shocks, the resulting model will be SVAR. We assume that the economy is described by a structural form equation:


(1) $$\text{f}\left(\text{x}\right)={\text{a}}_{0}+\sum_{\text{n}=1}^{\text{∞}}\left({\text{a}}_{\text{n}}\text{cos}\frac{\text{n}\text{π}\text{x}}{\text{L}}+{\text{b}}_{\text{n}}\text{sin}\frac{\text{n}\text{π}\text{x}}{\text{L}}\right)$$

Where *Y*_*t*_ = (*Y*_1*t*_,*Y*_2*t*, …………_*Y*_*nt*_) is *n* × 2 vector of endogenous variables, while *Y*_*t*−1_, is the corresponding lag terms of order i, ∅_*i*_ is the *n* × *n* matrix of autoregressive coefficients of vector *Y*_*t*−1_, for *i* = 1, 2, …... *p*.*c* = (*c*_1_,*c*_2, …………_*c*_*n*_) is the *n* × 1 intercept vector of the VAR model. ε_*t*_ = (ε_1*t*_, ε_2*t*_, ………ε_*nt*_) is the *n* × 1 vector of White Noise Process.

The variables considered in this study are crude oil prices, GDP, inflation rate, interest rate, and exchange rate.

## Empirical result

The statistical findings of the VAR model, as explained in the preceding part, are the Granger causality test, the impulse-response function and variance decomposition analysis.

### Unit root test

The present study applied the Augmented Dickey–Fuller (ADF) Test to deduct the unit root in the data. Table 2 shows the ADF results, specifying that all the variables are stationary at 1st difference.

**Table 2:** Unit root test.

**Augmented Dickey Fuller (1st difference)**
**Afghanistan**	**Variables**	**Statistics**	**Probabilities**
	Crude oil price	−4.262430	0.0041
	GDP	−6.198269	0.0001
	Inflation rate	−5.131361	0.0008
	Interest rate	−5.718421	0.0004
	Exchange rate	−3.145507	0.0400
Bangladesh	Crude oil price	−4.262430	0.0041
	GDP	−3.897833	0.0092
	Inflation rate	−5.130685	0.0008
	Interest rate	−4.202000	0.0046
	Exchange rate	−3.887091	0.0089
Bhutan	Crude oil price	−4.262430	0.0041
	GDP	−5.441077	0.0004
	Inflation rate	−6.842858	0.0000
	Interest rate	−6.334976	0.0001
	Exchange rate	−3.542284	0.0181
India	Crude oil price	−4.262430	0.0041
	GDP	−4.332428	0.0038
	Inflation rate	−3.556829	0.0176
	Interest rate	−5.824226	0.0002
	Exchange rate	−3.542284	0.0181
Maldives	Crude oil price	−4.262430	0.0041
	GDP	−4.729547	0.0021
	Inflation rate	−4.657452	0.0018
	Interest rate	−7.830043	0.0000
	Exchange rate	−3.105893	0.0432
Nepal	Crude oil price	−4.262430	0.0041
	GDP	−6.119441	0.0001
	Inflation rate	−5.002515	0.0010
	Interest rate	−4.206540	0.0049
	Exchange rate	−3.418297	0.0233
Pakistan	Crude oil price		
	GDP	−4.157490	0.0051
	Inflation rate	−5.364057	0.0004
	Interest rate	−8.263941	0.0000
	Exchange rate	−3.353446	0.0274
Sri Lanka	Crude oil price	−4.262430	0.0041
	GDP	−3.815342	0.0115
	Inflation rate	−6.085404	0.0001
	Interest rate	−10.25934	0.0000
	Exchange rate	−4.261104	0.0041

*H*_0_. The series of variables has a unit root.

### Granger causality test

To examine the causal relationship of the variables, the present study performed the Pairwise Dumitrescu Hurlin Panel Causality Test. The focus was on the causal relationship between the crude oil price and GDP, inflation rate, interest rate, and exchange rate of the South Asian countries that have experienced a series of reforms in crude oil pricing mechanism resulting in the significant changes. Thus, the correlation between the world oil prices and South Asian countries' domestic prices may have also changed considerably before and after the reforms. The result of the Table 3 shows that as an oil-importing country's dependence on outside oil has increased dramatically during the last decades, such as a 50% increase in 2007, and at the same time, the reforms of the oil-pricing mechanism have made the domestic oil price be more and correlated with the world oil price. Therefore, the crude oil price has been significantly affecting the South Asian economies which is consistent with the previous findings (Sharma et al., 2018; Zakaria et al., 2021).

**Table 3:** Pairwise Granger causality test.

**Null hypothesis**	**Obs**	***F*-statistic**	**Prob**.
Exchange rate does not Granger cause COP	160	1.48679	0.2245
COP does not Granger cause exchange rate	5.26667	0.0231
GDP does not Granger cause COP	160	4.91086	0.0281
COP does not Granger cause GDP	0.03253	0.8571
Inflation rate does not Granger cause COP	160	0.84317	0.3599
COP does not Granger cause inflation rate	1.99342	0.1600
Interest rate does not Granger cause COP	160	4.57672	0.0340
COP does not Granger cause interest rate	0.20790	0.6491
GDP does not Granger cause exchange rate	160	0.92700	0.3371
Exchange rate does not Granger cause GDP	5.30308	0.0226
Inflation rate does not Granger cause exchange rate	160	2.10165	0.1491
Exchange rate does not Granger cause inflation rate	1.83168	0.1779
Interest rate does not Granger cause exchange rate	160	0.01512	0.9023
Exchange rate does not Granger cause interest rate	0.09794	0.7547
Inflation rate does not Granger cause GDP	160	1.18647	0.2777
GDP does not Granger cause inflation rate	0.68173	0.4102
Interest rate does not Granger cause GDP	160	1.78318	0.1837
GDP does not Granger cause interest rate	0.39920	0.5284
Interest rate does not Granger cause inflation rate	160	2.56364	0.1114
Interest rate does not Granger cause interest rate	1.17242	0.2806

### Impulse-response functions

**Figure 1:**
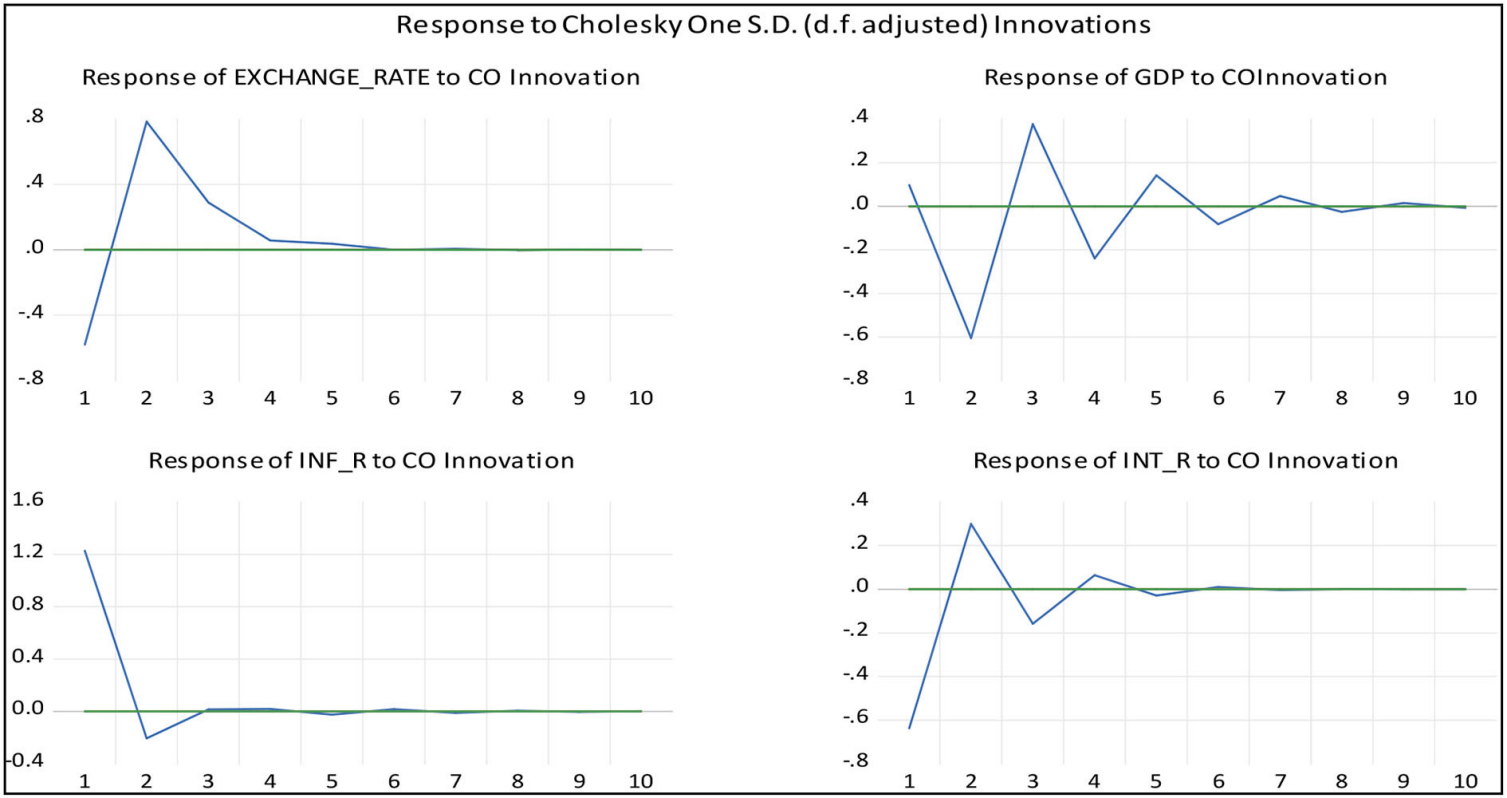
Impulse response function.

The impulse response function test was used to investigate the dynamic impact of crude oil price shocks on the VAR system. Figure 1 plots the responses of GDP, inflation rate, interest rate, and exchange rate to one unit innovation of the world crude oil price with two standard error bands. The impulse response function shows that exchange-rate negatively responds to crude oil prices in the 1st year and it takes almost 5 years to observe the oil price shocks. Similarly, GDP per capita is also severely affected by crude oil price shock and it responds in a monotonic fashion and it took 8 years to completely stabilize after oil shocks. Moreover, the interest rate, exchange rate, and inflation of the selected economies are also largely influenced by oil price movement in the initial phase, however; comparatively, it takes less time to absorb the shocks. It can be concluded from the impulse response function that all four macroeconomic variables of the South Asian counties are closely linked with oil price movements and their economies hugely rely on crude oil consumption. Hence, our findings confirm the previous research findings that crude oil prices significantly influence the macro-economic factors such as interest rate, price level, and industrial production (Ratti and Vespignani, 2016).

### Variance decomposition

Variance decomposition separates the variation in an endogenous variable into the component shocks to the model and provides information about the relative importance of each random innovation in affecting the variables in the VAR model.

The findings of variance decomposition for real GDP, inflation, interest rate, and real exchange rate over ten-year horizons are presented in Table 4. Following the purpose of this study to examine oil price shocks and their impact on GDP, inflation, interest rate, and exchange rate, the discussion is limited to the forecast error variance when crude oil price changes. In the case of South Asian economies, the estimated impacts demonstrate that oil price shocks explain unlike variations in the variables under consideration. The shock of crude oil price accounts for GDP, 3.98–2.52%, inflation rate 10.98–20.24%, interest rate 0.66–0.88%, and exchange rate 3.22–16.34% variation over the period of the ten-time horizon in the case of Afghanistan. Similarly, in Bangladesh, variations in GDP, inflation rate, interest rate, and exchange rate because of crude oil price shocks are 1.02–1.96, 4.22–10.82, 2.98–1.11, 0.31–40.71%, respectively, during the period of the ten-time horizon. These results are in line with previous research that oil prices have a significant impact on the interest and exchange rate of Bangladesh (Das and Dutta, 2020). Hence, it indicates that variation in crude oil price significantly influences the macroeconomic variables over the long period.

**Table 4:** Variance decomposition of crude oil price.

**Afghanistan**	**Periods**	**GDP**	**Inflation rate**	**Interest rate**	**Exchange rate**
	1	3.983422	10.98752	0.664534	3.224981
	2	4.240999	11.41795	0.752846	3.754973
	3	3.942463	10.81899	0.574172	12.15456
	4	3.258382	19.20028	0.594202	14.16525
	5	2.585530	17.52767	0.593104	15.73348
	6	2.241544	20.46851	0.844410	14.94151
	7	2.178864	20.66083	0.898576	14.21852
	8	2.228867	21.16323	0.922045	14.11017
	9	2.376429	20.96222	0.912963	14.74957
	10	2.521058	20.24301	0.882149	16.34150
Bangladesh	1	1.0204523	4.227654	2.980342	0.317895
	2	1.036953	4.566891	3.039595	0.341930
	3	0.787130	8.439812	9.477830	0.318571
	4	0.714841	13.24046	11.44906	2.640583
	5	1.734307	13.44597	10.50937	7.913017
	6	3.097125	12.41768	9.856907	10.80292
	7	3.246764	11.90370	9.403214	11.29189
	8	3.149368	11.54530	9.142191	11.37514
	9	3.088991	11.35410	9.048428	11.80840
	10	3.049892	11.14172	8.868823	12.79635
Bhutan	1	0.956735	0.534870	2.567234	8.549874
	2	0.961461	0.547906	3.032674	8.621670
	3	2.895036	0.462530	14.22526	8.799288
	4	3.049136	0.757089	23.16797	8.694187
	5	2.914345	1.316104	22.75732	15.48300
	6	2.907116	1.234222	21.21088	20.28804
	7	2.971778	1.206814	20.43093	22.23470
	8	3.174758	1.155924	19.09397	24.06217
	9	3.235599	1.055453	17.57333	25.40345
	10	3.125520	0.975407	16.59991	25.74172
India	1	20.97857	30.44578	0.235678	2.118984
	2	21.78856	31.43647	0.244117	2.146595
	3	16.24406	42.14653	14.59894	0.930729
	4	16.00508	38.34833	14.42043	5.429623
	5	18.83122	37.74932	13.85668	5.413247
	6	18.72344	36.35394	13.46522	6.063655
	7	18.95661	35.76032	13.31705	6.713571
	8	18.83437	35.62722	13.36270	7.630427
	9	18.77539	35.85933	13.28942	7.810686
	10	18.08024	36.73577	13.33390	8.499269
Maldives	1	0.523577	1.564324	1.895687	0.912487
	2	0.646132	1.481547	1.983574	0.924418
	3	0.959168	4.800460	3.346336	0.951689
	4	1.072759	5.735804	3.448491	1.338980
	5	1.135826	5.448410	3.300342	5.193609
	6	1.477947	5.133702	3.081523	10.76123
	7	2.304771	4.857119	2.940424	14.69392
	8	3.092099	4.653976	2.934544	17.30284
	9	3.472810	4.530506	2.954075	19.61292
	10	3.678377	4.485870	2.949252	21.70106
Nepal	1	2.454653	1.435681	0.657854	5.245436
	2	2.945779	1.445884	0.715931	5.370952
	3	2.515773	13.63195	0.722239	4.579056
	4	2.133640	19.35673	1.914377	8.677989
	5	2.063376	17.08075	1.643945	20.80387
	6	1.935101	15.80681	1.485973	28.04264
	7	1.906743	15.62143	1.425329	32.50176
	8	2.130673	14.12233	1.369307	35.29615
	9	2.078778	12.29148	1.252736	38.14521
	10	1.967736	10.82160	1.119896	40.71877
Pakistan	1	8.672853	6.567944	2.467810	3.435578
	2	8.892763	6.923038	2.654270	4.810249
	3	7.406789	14.74984	2.281677	3.953991
	4	8.869453	22.39503	1.527853	9.379938
	5	7.839039	20.93443	1.385257	13.29334
	6	8.924412	20.37495	1.465311	13.17527
	7	8.752046	19.62547	1.429102	16.58153
	8	8.853835	19.38702	1.392020	18.00221
	9	8.994708	19.04502	1.394795	18.43901
	10	8.676950	19.10024	1.345983	20.07478
Sri Lanka	1	12.37029	11.25705	5.188723	1.312227
	2	24.79700	8.192287	6.222766	20.04905
	3	28.45030	6.302620	6.057457	29.47177
	4	28.74200	8.687876	5.568560	30.68110
	5	27.94722	13.72839	5.070062	29.13573
	6	28.00526	15.02561	4.919427	28.65105
	7	27.99751	14.89658	4.907159	29.00192
	8	27.73992	14.92903	4.877352	29.41621
	9	27.51739	14.82316	4.840392	29.59879
	10	27.16289	15.01458	4.802698	29.46486

However, oil price shock during the whole period of 10 horizons in Bhutan explains about 0.95–3.12, 0.53–0.97, 2.56–16.59, and 8.54–25.74% of forecast error variation in GDP, inflation rate, interest rate, and exchange rate, respectively. Similarly, 20.97–18.08% variance in the GDP, 30.44–36.73% in the inflation rate, 0.23–36.73% in the interest rate and 2.11–8.49% in the exchange rate in India are due to crude oil price shock over a ten-year period. While, the crude oil price shock induces around 0.52–3.67% of the variation in the GDP of Maldives and it also explains about 1.56–4.48% of fluctuation in the inflation rate, 1.89–2.94% variation in the interest rate and 0.91–21.70% variation in the exchange rate during the entire ten-year horizon. Similarly, 2.45–1.96% variation in the GDP, 1.43–10.82% variation in the inflation rate, 0.65–1.11% variation in the interest rate and 5.24–40.71% variation in the exchange rate were explained throughout the 10 years horizon in Nepal.

The crude oil price shock during the ten-year horizon in Pakistan explains about 8.63–8.67% in the GDP, 6.56–19.10% in the inflation rate, 2.46–1.34% in the interest rate, 3.43–20.07% in the exchange rate, and are largely consistent with that of Jawad (2013). At last, crude oil price shocks induce around 12.37–27.16% of the variation in GDP in Sri Lanka and it explains about 11.25–15.01% of fluctuations in the inflation rate during the ten-year time horizon. The crude oil price shock also accounts for 5.18–4.80%, 1.31–29.46% variance in the interest rate and exchange rate, respectively, throughout the entire horizon. Overall, these findings confirm the previous findings that oil price shock significantly influence the macro-economic indicators of the SAARC countries specifically real GDP, interest rate, inflation, and exchange rate (Ahmed et al., 2019).

Moreover, the impulse response and especially variance decomposition effects ratify that crude oil shocks have a significant impact on GDP, inflation rate, interest rate, and exchange rate almost in the South Asian countries. The possible reason for that may be these countries are unable and incompetent in their exports. However, any negative impact of crude oil price on GDP, inflation rate, interest rate, and exchange rate may cause effect their export demand to decrease. Moreover, these countries may also be unable to attract foreign direct investment (FDI), which leads to minimized investment and ultimately a fall in aggregate output. Therefore, this research study suggests that oil price shocks have both short- and long-term impacts on macroeconomic variables of the South Asian countries.

## Conclusion

The South Asian countries are high-oil–consuming economies, had a greater reliance on oil imports and mainly rely on the market-oriented oil price mechanisms. Therefore, the influence of crude oil prices on these economies remains significant. Using the method of VAR analysis, this study assessed the effect of crude oil prices on the South Asian economies based on annual data from 2000 to 2020. The VAR approach is one of the most widely used techniques in the existing literature. The Granger causality test, impulse-response functions, and variance decomposition tools are used to explore the relationship between oil price volatility and macroeconomics indicators.

The Granger causality tests revealed that the South Asian economies have little impact on global oil prices. However, their high oil consumption makes them an important player in the global oil market, but they have not yet obtained influencing power in the global oil market. While on contrary, the global oil prices successfully affect the South Asian economy for the period under study.

The results of the impulse-response functions of the linear impact model demonstrate that the GDP of the South Asian countries is favorably correlated with world crude oil prices. An increase in crude oil prices should have increased production costs and consequently slowed GDP growth in oil-importing countries. Moreover, the result of the variance decomposition shows that crude oil shocks have a significant impact on GDP, inflation rate, interest rate, and exchange rate almost in all the South Asian countries because of incompetency in export, and a low level of foreign direct investment (Umar et al., 2020).

On the basis of the findings, it is suggested that change in public policies of South Asian countries in a way to reduce their dependency on oil energy and encourage them toward renewal and green energy sources by offering incentives and basic knowledge to relevant industries. This will not only reduce their oil dependency, but it will help to improve their environmental quality along with an increased profitability. Second, priority should be given to new/advanced technology for industrial production and higher taxes should be imposed on old technology to encourage industries for updated technology. This will ultimately improve production efficiency, reduce energy consumption, and enhanced aggregate economy with enlarged GDP.

This study focused only on single dimension that is the economic impact of crude oil, however, further study can investigate its impact on public health, life expectancy, and environmental degradation. It would be intriguing to explain the abnormal phenomenon both theoretically and empirically in future research.

## Data availability statement

The raw data supporting the conclusions of this article will be made available by the authors, without undue reservation.

## Author contributions

All authors listed have made a substantial, direct, and intellectual contribution to the work and approved it for publication.

## Conflict of interest

The authors declare that the research was conducted in the absence of any commercial or financial relationships that could be construed as a potential conflict of interest.

## Publisher's note

All claims expressed in this article are solely those of the authors and do not necessarily represent those of their affiliated organizations, or those of the publisher, the editors and the reviewers. Any product that may be evaluated in this article, or claim that may be made by its manufacturer, is not guaranteed or endorsed by the publisher.
